# miR-210 in Exosomes Derived from Macrophages under High Glucose Promotes Mouse Diabetic Obesity Pathogenesis by Suppressing NDUFA4 Expression

**DOI:** 10.1155/2020/6894684

**Published:** 2020-03-19

**Authors:** Feng Tian, Ping Tang, Zhilian Sun, Ruifen Zhang, Danhua Zhu, Junying He, Jixing Liao, Qinghua Wan, Jie Shen

**Affiliations:** ^1^Department of Endocrinology and Metabolism, The Third Affiliated Hospital of Shenzhen University, Shenzhen, Guangdong, China; ^2^Department of Endocrinology and Metabolism, The Third Affiliated Hospital of Southern Medical University, Guangzhou, Guangdong, China

## Abstract

**Objective:**

Type 2 diabetes mellitus (T2DM) is featured by insulin resistance and lipid metabolism dysregulation. A large number of miRNAs were identified in exosomes derived from adipose tissue macrophages associated with T2DM pathogenesis, but its pathogenic roles remain unknown. This study is aimed at investigating the function of miR-210 in diabetic obesity.

**Methods:**

Exosomes from mouse macrophage RAW264.7 cells were characterized by electron microscopy, combined with biomarker expression by western blot. Expression of miR-210 was determined by quantitative RT-PCR. Glucose uptake was measured by a fluorometric method, and the mitochondrial respiratory chain activity was evaluated by ELISA. The target gene of miR-210 was validated by dual-luciferase reporter and pull-down assays. A mouse obese diabetic model was established by a high-fat diet and streptozocin treatment.

**Results:**

miR-210 was highly expressed in exosomes derived from high glucose-induced macrophage RAW264.7 cells. Macrophage-derived exosomes impaired glucose uptake and mitochondrial CIV complex activity and suppressed NADH dehydrogenase ubiquinone 1 alpha subcomplex 4 (NDUFA4) expression in 3T3-L1 adipocytes. miR-210 directly bind with mRNA sequences of NDUFA4 gene. Inhibition of miR-210 mitigated the effects of macrophage-derived exosomes on the glucose uptake and complex IV (CIV) activity in 3T3-L1 adipocytes, and NDUFA4 overexpression offset the inhibition of glucose uptake and CIV activity by macrophage-derived exosomes. Furthermore, mice with miR-210 knockout showed greatly repressed diabetic obesity development.

**Conclusion:**

miR-210 derived from adipose tissue macrophages promotes mouse obese diabetes pathogenesis by regulating glucose uptake and mitochondrial CIV activity through targeting NDUFA4 gene expression.

## 1. Introduction

Type 2 diabetes mellitus (T2DM), also known as type 2 diabetes (T2D), is a common metabolic disease characterized by hyperglycemia and insulin resistance due to the lack of normally functioning pancreatic *β*-cells [1–3]. Epidemiological investigations showed that T2DM accounts for more than 90% of all diabetes cases, whose incidence has been rapidly increasing since 1960, in parallel with obesity, and almost 400 million patients are suffering from T2DM worldwide [1, 4]. Patients with T2DM usually suffer from weight loss and increased thirst, followed by severe complications such as cardiovascular disease, diabetic retinopathy, and kidney failure [1, 5]. Currently, T2DM has been regarded as a multifactorial disease, and its incidence was found to be associated with both genetic factors and lifestyles such as obesity due to high consumption of dietary fats, alcohol consumption, and smoking [6]. However, the molecular mechanisms underlying the pathogenic processes of T2DM have still not been fully understood.

T2DM pathogenesis was closely associated with dysfunction of adipose tissue macrophages (ATMs), especially the altered macrophage metabolism which usually causes adipose tissue inflammation and obesity [7]. Mitochondria dysfunction of ATMs has been well-documented as one critical cellular event during T2DM pathogenic processes [2, 3, 8]. The damages of mitochondria could result into alteration of the beta oxidation pathway and ATP production, as well as excessively accumulated reactive oxygen species (ROS) [3]. Mitochondria play critical roles in insulin release from pancreatic *β*-cells in response to glucose stimulation, while mitochondrial dysfunction causes decreases of fatty acid breakdown by a beta oxidation pathway and suppressed ATP production, which lead to beta cell dysfunction and insulin resistance, as well as cellular metabolism dysregulation [3]. Specifically, the impaired activity or assembly of the mitochondrial complex IV (CIV) was reported to induce fatty acid oxidation and promoted intracellular lipid accumulation in white adipocytes, mediating the age-dependent obesity [9]. However, the specific mechanisms of adipose tissue macrophages during T2DM pathogenesis still remain largely unexplored.

Exosomes are 40-100 nm sized small vesicles secreted by various cell types and involved in a number of biological processes and pathogenesis of human disorders such as myocardial ischemia/reperfusion injury, tumor progression, and various metabolic diseases [10–12]. Recent progresses in the diabetes mellitus research community disclosed that exosomes from different tissues or cell types were widely associated with the pathogenesis of diabetes mellitus and related complications [13, 14]. For instance, exosomes secreted by the bone marrow-derived mesenchymal stem cells were previously reported to improve diabetes-induced cognitive impairment while being transferred into the damaged neurons and astrocytes [13]. Recently, exosomes derived from macrophages in the adipose tissues were shown to regulate the sensitivity of adipocytes to insulin, thus associated with diabetic obesity pathogenesis [15]. Nevertheless, little is known about the underlying molecular events.

MicroRNA, abbreviated as miRNA, refers to a large group of small noncoding RNA molecules which usually contain about 22 nucleotides and widely expressed in various species including plants, animals, and even viruses [16]. It has been well established that miRNAs perform their pleiotropic biological and pathogenic roles by posttranscriptionally silencing expression of functional genes via base pairing with target mRNA molecules [17, 18]. Due to its potent capability of regulating functional gene expression, miRNA expression and functioning have been well-documented as critical players in cell growth, development, and pathogenesis of a number of human disorders such as various cancers, neurodegenerative disorders, and cardiovascular disease [19–22]. More importantly, miRNAs have also been revealed to be closely linked with obesity and pathogenesis of type 2 diabetes mellitus [1, 15, 23, 24]. The alteration of miRNA profiles in primary human islets and enriched beta-cells was recently shown to underlie the genetics of type 2 diabetes (T2D) susceptibility [25]. For instance, microRNA 144 could repress insulin signaling during type 2 diabetes mellitus development through suppressing the expression of Insulin Receptor Substrate 1 (IRS1) [26]. Currently, miR-210 has been reported to have significant contributions in various diseases including cardiovascular ischemic diseases [27, 28], cancer [29, 30], Graves' disease [31], and psoriasis [32]. Moreover, a group of miRNAs including miR-210 were differentially expressed in exosomes from obese adipose tissue macrophages [15]. However, its specific pathogenic roles during T2DM development have never been previously addressed.

In the present study, we aimed to investigate the effects of miR-210 on type 2 diabetic obesity mediated by regulation of functional gene expression, which would provide novel insights into microRNA-mediated T2DM pathogenesis.

## 2. Material and Methods

### 2.1. Cell Lines and Treatment

The mouse macrophage cell line RAW264.7 was obtained from the Cell Bank of the Chinese Academy of Sciences (Cat. No: TCM13; Shanghai, China) and cultured in Dulbecco's modified Eagle medium (DMEM) (#12800017, GIBCO) supplemented with 10% fetal bovine serum (FBS) and 1.5 g/L NaHCO_3_ at 37°C in a standard humidified cell culture chamber supplied with 5% CO_2_. Mouse 3T3-L1 adipocytes were cultured in DMEM with 10% fetal calf serum, 4 mM glutamine, 10 *μ*M dexamethasone, and 0.5 mM isobutylmethylxanthine. For high glucose treatment, RAW264.7 cells were cultured in DMEM medium containing 30 mm/L glucose (Sigma-Aldrich) for 24 h. The 3T3-L1 adipocytes cultured in 6-well plates were treated with 2 *μ*g exosomes for 24 h before functional analysis.

### 2.2. Obese Diabetic Mice Establishment

A total of 36 male C57BL/6JNju mice and miR-210^−/−^ (B6/JNju-Mir210^em1Cd235^/Nju, T000745) mice were purchased from Nanjing Biomedical Research Institute of Nanjing University. All mice (SPF grade) aged between 4 and 6 weeks with a weight of 20-22 g were randomly divided into the wild-type (WT), wild-type obese diabetes (WT-OD), and knockout obese diabetes (KO-OD) groups (12 mice/group). The procedures were approved by the Animal Ethics Committee of the Third Affiliated Hospital of Southern Medical University (Guangzhou, China). For the establishment of a diabetes mellitus animal model, wild-type or miR-210 knockout (KO) mice were fed a high-fat diet for approximately 7 weeks to induce obesity, which was defined by a body weight 20% higher than that of mice fed a normal diet. Subsequently, obese mice were fasted overnight and treated with streptozocin (STZ; 40 mg/kg, diluted in 0.1 M citrate buffer) by intraperitoneal injection. Mice in the WT group were fed a normal diet and injected intraperitoneally with an equal volume of normal saline. Seventy-two hours later, the successful establishment of the obese diabetic model (OD) was defined by a fasting blood sugar (FBS) of >16.6 mmol/L and an over 50% increase of urine volume during successive three measurements, combined with the glucose tolerance test as previously described [33]. Subsequently, mice were sacrificed by euthanasia with CO_2_, followed by body weight measurement and collection of abdominal brown adipose tissues which were then kept at -80°C for following assays.

### 2.3. Exosome Isolation from Macrophage Cells

The exosomes from mouse macrophages were extracted using the Exosome Extraction Kit (#BB-3901-1; BestBio, Shanghai, China) following the manufacturer's instructions.

Briefly, mouse RAW264.7 macrophage cells were cultured in DMEM supplemented with 10% exosome-depleted FBS (#A2720803; Thermo Fishier Scientific) for 72 hours. For induction with high glucose, cells were cultured in DMEM containing 30 mm/L glucose and 10% exosome-depleted FBS for 24 hours. Dead RAW264.7 cells and debris were removed by centrifugation at 3000 g for 15 min, followed by centrifugation at 10,000 g for 10 min. The supernatants were mixed with Extraction Buffer A, kept at 4°C overnight, and centrifuged at 10,000 g for 60 min. The pellets containing exosomes were then washed twice with and resuspended in exosome storage solution. The characterization of macrophage-derived exosomes was confirmed by electron microscopy and detecting exosome biomarkers TSG101, HSP70, and CD63 by western blotting.

## 3. Separation of Serum Exosomes

Serum exosome separation kit (Invitrogen, cat. no. 4478360) was applied to isolate serum exosomes based on the instruction provided by the supplier. Serum was extracted from mice in each group and centrifuged (2000 g for 30 min) to remove the serum precipitation. The supernatant was added with 10% volume exosome separation reagent at 4°C for 60 min. After centrifugation at 10,000 g for 10 min, the precipitations were dissolved and preserved at -80°C.

### 3.1. Electron Microscopy

The electron microscopy analysis of exosomes enriched from mouse macrophage cell supernatant was performed as previously described [15]. Briefly, exosomes were first fixed in 2% paraformaldehyde and loaded onto Formvar and carbon-coated copper grids, which were then incubated with 2% gelatin for 25 min at 37°C, washed with PBS containing 0.15 M glycine, blocked using 1% skin gelatin from cold water fish, and finally viewed and photographed by electron microscope (#HT7700, HITACHI).

### 3.2. Quantitative RT-PCR Assay

The mRNA levels of functional genes or miRNA levels in this study were determined by a quantitative RT-PCR method. Total RNA samples were extracted from cultured cells or mouse tissues using Trizol reagent (#9109; TAKARA) according to the manufacturer's instructions. The synthesis of cDNA library was finished using approximately 3.0 *μ*g total RNAs and the BestarTM qPCR RT kit (#2220; DBI) following the manufacturer's instructions. Relative mRNA or miRNA levels were finally detected using the BestarTM qPCR MasterMix (#2043; DBI) as instructed by the manufacturer. The quantitative RT-PCR assay was biologically repeated for more than three times, and U6 was applied as the internal standard. The expressional levels of gene and miRNA were finally quantitated using the standard 2-*^ΔΔ^*Ct method. All sequences of primers used for quantitative RT-PCR assay in this study are presented in Table 1.

### 3.3. Western Blotting

Protein abundances in cell lines and mouse brown adipose tissues were analyzed by western blotting using specific antibodies in this study. Briefly, total proteins from cells, exosomes, or mouse brown adipose tissues were extracted using the Western & IP Cell Lysis Buffer (#P0013; Beyotime, Shanghai, China) following the manufacturer's instructions. Protein concentration was determined using the Thermo Pierce 23227 BCA Protein Assay Kit 500 mL (#23227; Thermo Fishier Scientific). About 30 *μ*g total proteins from each sample were loaded and separated by 12% SDS-PAGE, transferred to PVDF membranes, which was subsequently blocked with 5% fat-free milk solution, incubated with primary antibodies overnight at 4°C, followed by incubation with appropriate secondary antibodies and final development with enhanced chemiluminescence substrate kit (#KLS0500, Merck Millipore). GAPDH (glyceraldehyde-3-phosphate dehydrogenase) was included as the internal standard. The primary antibodies used for protein abundance determination were listed as follows: anti-NDUFA4 antibody (#ab129752; Abcam), anti-VDAC1 (#ab15895; Abcam), anti-TSG101 (#BM4821; Boster Biological Technology, Wuhan, China), anti-CD63 (#ab217345; Abcam), anti-HSP70 (#ab2787; Abcam), and anti-GAPDH (#ab8245; Abcam). Protein level determination was done by at least three biological repeats.

### 3.4. Glucose Uptake Assay

Glucose uptake by 3T3-L1 cells was determined using the Screen Quest™ Fluorimetric Glucose Uptake Assay Kit (#36500; AAT Bioquest, USA) following the manufacturer's instructions. Briefly, 3T3-L1 cells seeded in 96-well plates (80,000 cells/well) were incubated with 10 *μ*L/well 2-DG (2-deoxyglucose) for 30 min at 37°C, lysed with acidic lysis buffer (25 *μ*L/well) for 20 min at 37°C, mixed with 2-DG Uptake Assay working solution (50 *μ*L/well) followed by incubation for 60 min at room temperature, and the glucose uptake was finally determined by monitoring the fluorescence increase at Ex/Em = 540/590 nm using a fluorescence microplate reader. At least three biological replicates were performed for statistical analysis.

### 3.5. ELISA

The mitochondrial CIV activity in 3T3-L1 cells was detected by the Enzyme-Linked Immunosorbent Assay (ELISA) using the Mitochondrial Respiratory Chain CIV Complex Activity Kit (#BC0940; Solarbio, Beijing, China) following the instructions by the manufacturer. Briefly, approximately 5∗10^6^ 3T3-L1 cells were lysed in 1 mL CIV Complex Extraction Buffer, followed by centrifugation at 600 g for 10 min at 4°C. The supernatant was collected and centrifuged at 11000 g for 15 min at 4°C, and the CIV complex in the precipitates was then resuspended in 400 *μ*L CIV Complex Extraction Buffer and disrupted by sonification. The CIV complex activities in cells were finally evaluated by measuring the OD_550_ values. The ELISA was biologically repeated for at least three times.

### 3.6. Cell Transfection

To silence miR-210 expression, the miR-210 inhibitor and negative control were synthesized by the GenePharma Company (Shanghai, China) and transfected into RAW264.7 cells using the Lipofectamine 2000 reagent (Invitrogen, USA) according to the manufacturer's instructions. Meanwhile, exosomes were extracted from the transfected RAW264.7 cells, and the exosomes were then applied to treat 3T3-L1 cells.

For overexpression of NDUFA4, the coding sequences were amplified by PCR using the following primers: Ndufa4-F1: 5′-GGGGTACCATGCTCCGCCAGATCCTCGG-3′ and Ndufa4-R1: 5′-CCGCTCGAGTTAGAAGTCTGGGCCTTCTTTCTTCAGT-3′. The obtained NDUFA4 gene sequences were ligated with pcDNA3.0 plasmid and transfected into the 3T3-L1 adipocytes using the lipofectamine 2000 reagent as described above. The overexpression of NDUFA4 was finally confirmed by quantitative RT-PCR and western blotting.

### 3.7. Dual-Luciferase Reporter Assay

The psiCHECK-NDUFA4-WT plasmid and the psiCHECK-NDUFA4-MUT plasmid were purchased from the Vipotion Company (Guangzhou, China). The miR-210 mimics and the negative control were synthesized by GenePharma Company (Shanghai, China). These plasmids and sequences were cotransfected into 3T3-L1 cells as designated following the manufacturer's instructions. Forty-eight hours after cell transfection, cells were lysed using the PLB (Passive Lysis Buffer) and detected using the GloMax® 20/20 Luminometer (Promega). At least three biological repeats were performed for statistical analysis.

### 3.8. Pull-Down Assay

miR-210 NC and miR-210 were biotinylated to be bio-NC and bio-miR-210 probes by GenePharma Company (Shanghai, China). RAW264.7 cells were transfected with Bio-NC and bio-miR-210 probes for 48 h. The treated cells were lysed using lysis buffer and incubated with Dynabeads M-280 Streptavidin (Invitrogen) by referring to the experimental instructions. Beads were applied to incubate the biotinylated miR-210 for 10 min. After washing, the bound RNAs were examined by RT-PCR assay.

### 3.9. Statistical Analysis

Quantitative data in this study were presented as the mean ± SEM, and statistical analysis was finished with combined application of the GraphPad Prism 5.0 and SPSS 18.0 software. The significance of differences between two or more groups was tested by Student's *t*-test or one-way analysis of variation (ANOVA) as appropriate. Significant differences were finally defined by a *P* value of <0.05.

## 4. Results

### 4.1. High Expression of miR-210 in Exosomes Secreted by Mouse Macrophages under High Glucose

**Figure 1:**
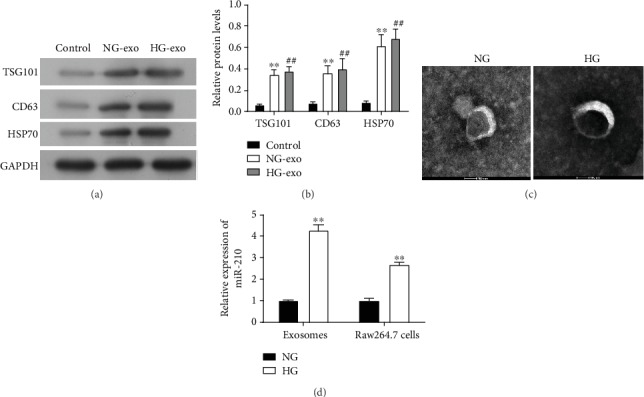
Extraction and characterization of exosomes from mouse macrophage under high glucose treatment. (a, b) Protein abundances of HSP70, CD63, and TSG101 in exosomes extracted from Raw264.7 cells treated with high glucose. Protein abundances were determined by western blotting (a) and quantitated for statistical analysis (b). The remaining supernatant of Raw264.7 cell culture following exosome extraction was used as the control group. GAPDH was used as the internal standard. (c) Observation of exosomes from mouse macrophage under high glucose treatment by electron microscopy. Bar = 100 nm. (d) miR-210 levels in exosomes derived from Raw264.7 cells treated with high glucose. The miR-210 levels were measured by quantitative RT-PCR levels. NG-exo: normal glucose exosomes; HG-exo: high glucose exosomes; HSP70: heat shock protein 70; TSG101: tumor susceptibility gene 101; GAPDH: glyceraldehyde-3-phosphate dehydrogenase; ^∗^*P* < 0.05 and ^∗∗^*P* < 0.01 (compared with the control group); ^##^*P* < 0.01 (compared with the normal glucose exosome group).

For the analysis of the involvement of macrophage-derived miR-210 in diabetic obesity, we first cultured the mouse macrophage Raw264.7 cells and extracted the exosomes from the supernatant of cell culture after being treated with high glucose (30 mg/mL) for 24 h. We analyzed the protein abundances of three major exosome biomarkers HSP70 (heat shock protein 70), CD63, and TSG101 (tumor susceptibility gene 101), which were all shown to significantly elevate in exosomes derived from Raw264.7 cells treated with or without high glucose, in comparison with those in Raw264.7 cells under normal culture (Figures 1(a) and 1(b)). The extracted exosomes from mouse macrophages were first conformed by observation through electron microscopy, showing an exosome diameter of approximately 150 nm (Figure 1(c)). Subsequently, we detected the expressional levels of miR-210 in both the Raw264.7 cells and the extracted exosomes and found that miR-210 levels were greatly increased by treatment with high glucose in both mouse macrophage cells and exosomes derived from them (Figure 1(d)). Also, higher levels of miR-210 were observed in exosomes compared with the Raw264.7 cells (Figure 1(d)). These results showed that we successfully extracted exosomes from mouse macrophages and miR-210 was highly expressed in exosomes from macrophages under high glucose.

### 4.2. Exosomes Derived from High Glucose-Induced Macrophages Suppressed Glucose Uptake, Complex IV Activity, and NDUFA4 Expression in Adipocytes

**Figure 2:**
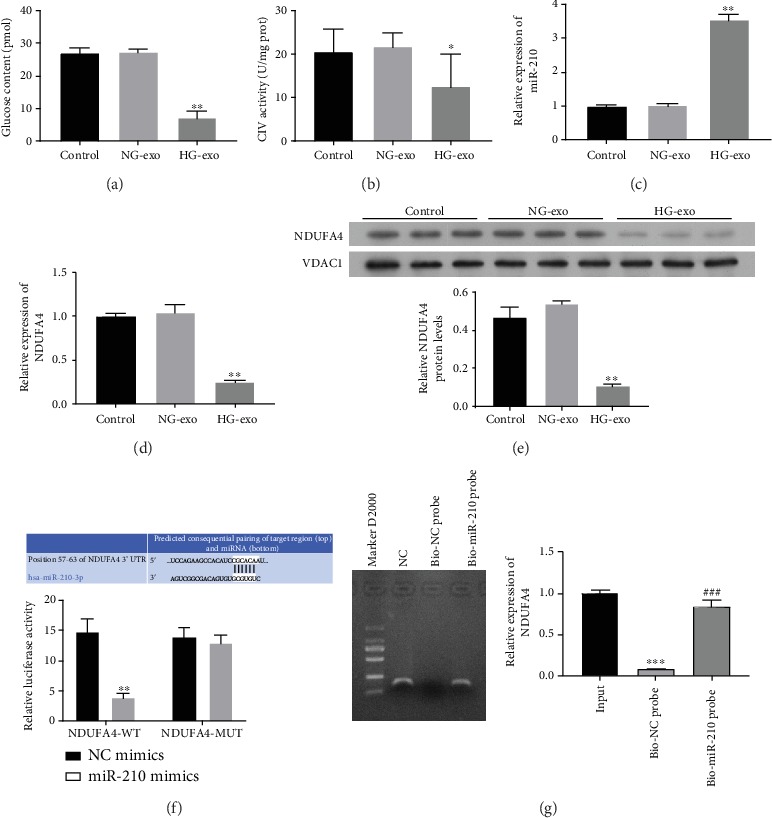
Suppression of adipocyte glucose uptake, CIV activity, and NDUFA4 expression by macrophage-derived exosomes. (a) Glucose uptake by 3T3-L1 cells suppressed by macrophage-derived exosomes under high glucose treatment. Glucose content in 3T3-L1 cells was analyzed by the fluorometric method. (b) Mitochondrial respiratory chain complex IV activity in 3T3-L1 cells treated with exosomes derived from macrophages under high glucose treatment. ELISA was performed to analyze CIV activity. (c, d) Expression levels of miR-210 (c) and NDUFA4 gene (d) in 3T3-L1 cells treated with exosomes derived from macrophages. miR-210 expression and NDUFA4 expression were determined by quantitative RT-PCR. (e) NDUFA4 protein levels in 3T3-L1 cells treated with exosomes derived from macrophages by western blotting. VDAC1 was used as the internal standard. The 3T3-L1 cells cultured under normal conditions were used as the control group in above assays. (f) The predicted binding of miR-210 with 3′-UTR regions of NDUFA4 gene. Targeting NDUFA4 gene by miR-210 in 3T3-L1 cells confirmed by dual-luciferase reporter assay. (g) The possible interaction between miR-210 and NDUFA4 was examined by pull-down assay using bio-miR-210 probes, and the relative expression of NDUFA4 was quantified. NG-exo: normal glucose exosomes; HG-exo: high glucose exosomes; CIV: complex IV; NDUFA4: NADH dehydrogenase (ubiquinone) 1 alpha subcomplex 4; VDAC1: voltage-dependent anion channel 1; WT: wild type; MUT: mutant; NC: negative control; ^∗^*P* < 0.05 (compared with the normal glucose exosome group); ^∗∗^*P* < 0.01 (compared with the normal glucose exosome or NDUFA4-WT+NC group); ^∗∗∗^*P* < 0.01 (compared with the input group); ^###^*P* < 0.01 (compared with the bio-NC probe group).

To investigate the effects of macrophage-derived exosomes on adipocyte functions, exosomes extracted from RAW-264.7 cells treated with or without high glucose were used to incubate with 3T3-L1 adipocytes. We found that exosomes from RAW-264.7 cells under high glucose treatment greatly suppressed the glucose uptake in 3T3-L1 adipocytes, compared with those under normal glucose (Figure 2(a)). Moreover, the mitochondrial respiratory chain complex IV (CIV) activity in 3T3-L1 adipocytes was also remarkably suppressed by exosomes from RAW-264.7 cells under high glucose treatment (Figure 2(b)). These results showed the effective suppression of glucose uptake and mitochondrial function by macrophage-derived exosomes. In addition, miR-210 expression in 3T3-L1 adipocytes was significantly elevated by incubation with exosomes from high glucose-treated macrophages (Figure 2(c)).

NADH dehydrogenase (ubiquinone) 1 alpha subcomplex 4 (NDUFA4) is one component of mitochondrial CIV complex involved in mitochondrial dysfunction in syndromic obesity and diabetes [34–36]. We found that NDUFA4 gene expression was markedly downregulated by incubation with exosomes from high glucose-treated macrophages, compared with 3T3-L1 adipocytes treated with exosomes from RAW-264.7 cells under normal culture (Figure 2(d)). Moreover, the suppressed NDUFA4 expression in 3T3-L1 cells by exosomes from macrophage under high glucose was also confirmed by western blotting (Figure 2(e)). By TargetScan7.2 software, the 3′-UTR region of NDUFA4 gene was predicted to be a potential target gene of miR-210 (Figure 2(f)). More importantly, we showed by dual-luciferase reporter assay that miR-210 mimics induced a significant decrease of luciferase signal in 3T3-L1 cells, which was abolished by mutation of the 3′-UTR region of NDUFA4 gene, showing that miRNA-210 could directly bind with NDUFA4 gene (Figure 2(f)). Besides, pull-down assay was carried out to identify the interaction between miR-210 and NDUFA4. As displayed in Figure 2(g), the expression of NDUFA4 was dramatically increased in the bio-miR-210 probe group with respect to the bio-NC probe group, indicating that there is interaction between miR-210 and NDUFA4. These results revealed that exosomes derived from macrophage under high glucose suppressed NDUFA4 gene expression in adipocytes, which could be targeted by miR-210.

### 4.3. miR-210 in Exosomes Derived from High Glucose-Induced Macrophages Suppressed Adipocyte Glucose Uptake and Mitochondrial Function Targeting NDUFA4 Expression

**Figure 3:**
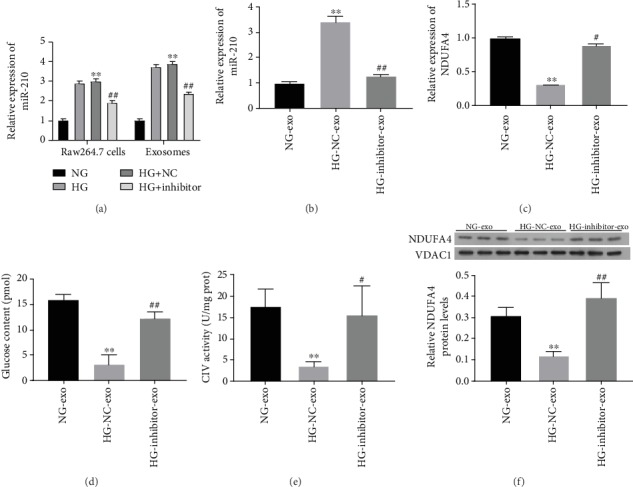
miR-210 in exosomes derived from macrophages under high glucose inhibited adipocyte glucose uptake, CIV activity, and NDUFA4 expression. (a) Identification of miR-210 expression by RT-PCR assay in the treated Raw264.7 cells and exosomes derived from macrophages. Expression levels of miR-210 (b) and NDUFA4 gene (c) in 3T3-L1 cells treated with exosomes extracted from RAW-264.7 cells under high glucose after miR-210 inhibitor. miR-210 and NDUFA4 mRNA levels were analyzed by quantitative RT-PCR. (d) Glucose uptake by 3T3-L1 cells treated with exosomes from RAW-264.7 cells treated with miR-210 inhibitor under high glucose. Glucose content in 3T3-L1 cells was determined by the fluorometric method. (e) CIV activity in 3T3-L1 cells incubated with exosomes from RAW-264.7 cells with miR-210 inhibitor under high glucose. The ELISA method was used to analyze CIV activity. (f) NDUFA4 protein abundances in 3T3-L1 cells treated with exosomes derived from RAW-264.7 cells with silenced miR-210 expression under high glucose. VDAC1 was used as the internal standard. NG: normal glucose; HG: high glucose; NC: negative control; exo: exosomes; CIV: complex IV; NDUFA4: NADH dehydrogenase (ubiquinone) 1 alpha subcomplex 4; VDAC1: voltage-dependent anion channel 1; ^∗∗^*P* < 0.01 (compared with the NC-normal glucose group); ^#^*P* < 0.05 and ^##^*P* < 0.01 (compared with the NC-high glucose group).

To explore the effects of miR-210 in exosomes from macrophages under high glucose on adipocyte functions, firstly, we identified the level of miR-210 in the treated Raw264.7 cells and exosomes derived from macrophages. Our results found that the level of miR-210 was upregulated in the HG group relative to the NG group, while the miR-210 inhibitor could weaken this upregulation induced by HG in Raw264.7 cells and exosomes derived from macrophages (Figure 3(a)). Afterwards, we treated the macrophages with the miR-210 inhibitor, followed by exosome extraction and incubation with the adipocytes. We proved that miR-210 inhibitors also increased NDUFA4 expression in 3T3-L1 cells incubated with exosomes from RAW-264.7 cells under high glucose, while miR-210 expression showed opposite alterations (Figures 3(b) and 3(c)). More importantly, we found that the suppressed glucose uptake and CIV activity in 3T3-L1 cells by exosomes from RAW-264.7 cells under high glucose were greatly recovered by miR-210 inhibitors (Figures 3(d) and 3(e)). In consistence, the upregulation of NDUFA4 expression in 3T3-L1 cells treated with exosomes from RAW-264.7 cells by miR-210 inhibitors was also confirmed by western blotting (Figure 3(f)). These results showed the suppression of adipocyte glucose uptake and CIV activity by exosomes from RAW-264.7 cells under high glucose was mediated by miR-210 which suppressed NDUFA4 expression in adipocytes.

### 4.4. NDUFA4 Overexpression Mitigated Suppression of Adipocyte Glucose Uptake and Mitochondrial Function by Exosomes Derived from Macrophage under High Glucose

**Figure 4:**
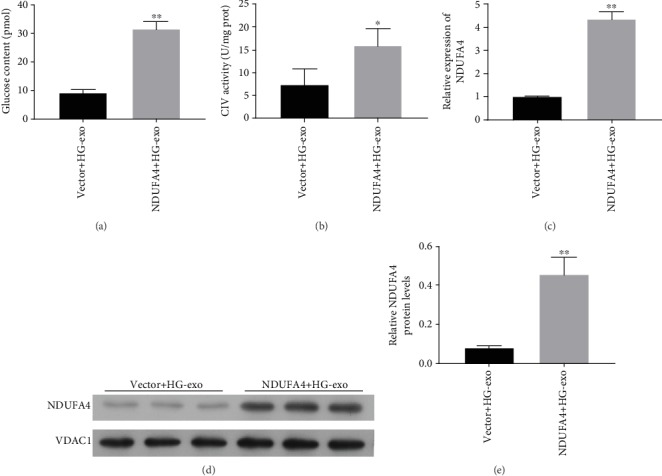
NDUFA4 overexpression counteracted the inhibition of adipocyte glucose uptake and CIV activity by exosomes derived from macrophages under high glucose. (a) Glucose uptake in NDUFA4-overexpressing 3T3-L1 cells treated with exosomes from RAW-264.7 cells under high glucose treatment. Glucose levels in 3T3-L1 cells were measured by the fluorometric method. (b) CIV activity in NDUFA4-overexpressing 3T3-L1 cells treated with exosomes derived from RAW-264.7 cells under high glucose treatment. (c) NDUFA4 mRNA levels in NDUFA4-overexpressing 3T3-L1 cells treated with exosomes extracted from RAW-264.7 cells under high glucose. The quantitative RT-PCR method was used to analyze NDUFA4 mRNA levels. (d) NDUFA4 protein levels in NDUFA4-overexpressing 3T3-L1 cells treated with exosomes derived from high glucose-induced RAW-264.7 cells. VDAC1 was used as the internal standard. NDUFA4: NADH dehydrogenase (ubiquinone) 1 alpha subcomplex 4; HG: high glucose; exo: exosomes; CIV: complex IV; ^∗^*P* < 0.05; ^∗∗^*P* < 0.01.

To analyze the roles of NDUFA4 gene in adipocyte function regulation by macrophage-derived exosomes, the NDUFA4 gene was overexpressed in 3T3-L1 cells. We observed that NDUFA4 overexpression in 3T3-L1 cells significantly abrogated the suppression of glucose uptake in 3T3-L1 cells by exosomes from macrophages under high glucose treatment (Figure 4(a)). Similarly, the inhibition of mitochondrial CIV complex in 3T3-L1 cells by exosomes from macrophages under high glucose was also greatly recovered by overexpression of NDUFA4 gene in 3T3-L1 cells (Figure 4(b)). Finally, the overexpression of NDUFA4 gene in 3T3-L1 cells was confirmed by both quantitative RT-PCR (Figure 4(c)) and western blotting (Figure 4(d)). These results showed that the suppression of glucose uptake and mitochondrial CIV activity in 3T3-L1 cells by exosomes derived from macrophages under high glucose was effectively mitigated by the overexpression of NDUFA4 gene in 3T3-L1 cells.

### 4.5. miR-210 Knockout Suppressed Diabetic Obesity by Increasing NDUFA4 Expression

**Figure 5:**
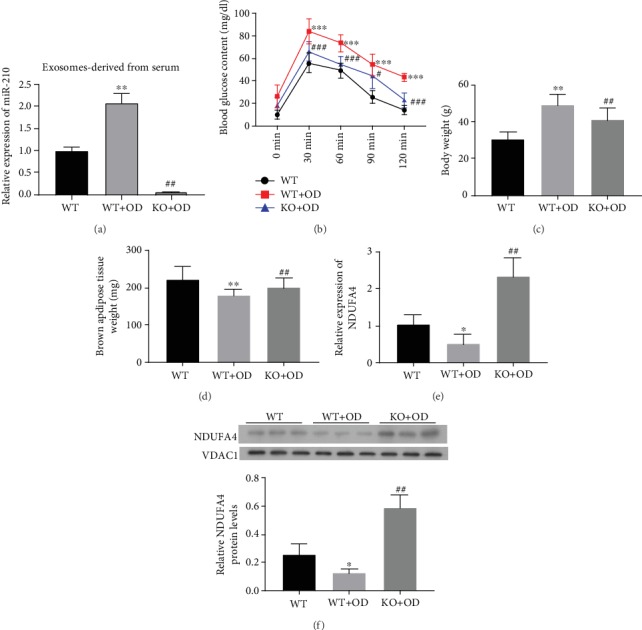
miR-210 knockout suppressed diabetic obesity progression and elevated NDUFA4 expression in a mouse model. (a) The level of miR-210 was determined by RT-PCR analysis in exosome derived from serum of miR-210 WT and KO diabetic obesity model mice. (b) Blood glucose content in the diabetic obesity model established using the miR-210 knockout mice. Glucose content in the mouse blood was analyzed by the fluorometric method. (c, d) Body weight (c) and the brown adipose tissue weight (d) were detected on the diabetic obesity model established using the miR-210 knockout mice. (e) NDUFA4 mRNA levels in the diabetic obesity model established using the miR-210 knockout mice. Quantitative RT-PCR was performed to analyze the NDUFA4 mRNA levels. (f) NDUFA4 protein abundances in the diabetic obesity model established using the miR-210 knockout mice by western blotting. VDAC1 was used as the internal standard. NDUFA4: NADH dehydrogenase (ubiquinone) 1 alpha subcomplex 4; VDAC1: voltage-dependent anion-selective channel 1; WT: wild type; KO: knockout; OD: obese diabetes. ^∗^*P* < 0.05 and ^∗∗^*P* < 0.01 (compared with the WT group); *P* < 0.01 (compared with the WT+OD group).

To further confirm the pathogenic roles of miR-210 in diabetic obesity pathogenesis, the miR-210 WT and knockout (KO) mice were used to establish the diabetic obesity by high-fat feeding and intraperitoneal injection of STZ. The results from RT-PCR identification showed that the level of miR-210 was highly expressed in the exosomes derived from serums of miR-210 WT diabetic obesity model mice compared with that in miR-210 WT mice, while the level of miR-210 was lowly expressed in the exosomes derived from serums of miR-210 KO diabetic obesity model mice compared with that in miR-210 WT diabetic obesity model mice (Figure 5(a)). In addition, we proved that compared with the wild-type (WT) mice, we found that the blood glucose levels were significantly increased in the diabetic obesity model established with WT mice as the time went on after STZ injection, which were then greatly suppressed in the diabetic obesity model established with miR-210 KO mice (Figure 5(b)). Moreover, the mouse weight of the model group was remarkably elevated but significantly downregulated by miR-210 knockout (Figure 5(c)). On the contrary, the brown adipose tissue weight in model mice was markedly decreased compared with that in the wild-type mice, which was then significantly elevated by miR-210 knockout (Figure 5(d)). In consistence with the above-introduced cellular assay results, the NDUFA4 expression was downregulated in the diabetic obesity mice, which was greatly recovered in the mouse model established using the miR-210 KO mice (Figure 5(e)). The upregulation of NDUFA4 expression by miR-210 knockout in mice tissues was also confirmed by western blotting (Figure 5(f)). Together, these assays further verified the critical roles of miR-210 in diabetic obesity pathogenesis, which was mediated by suppression of NDUFA4 expression.

## 5. Discussion

Adipose tissue macrophage dysfunction is involved in T2DM pathogenesis, which might be mediated by macrophage-derived exosomes [7, 15]. In the present study, we purified exosomes from high glucose-induced mouse macrophage RAW264.7 cells and revealed high miR-210 expression in these exosomes. Exosomes derived from macrophages under high glucose effectively inhibited glucose uptake and mitochondrial CIV complex activity and suppressed NDUFA4 expression in 3T3-L1 adipocytes. Moreover, we showed that miR-210 could directly bind with NDUFA4 gene sequence. Next, we proved that the effects of macrophage-derived exosomes on adipocytes could be mitigated by miR-210 inhibitors or NDUFA4 overexpression in adipocytes. Finally, we confirmed the prodiabetic obesity role of miR-210 by miR-210 knockout in the mouse model. These observations disclosed a new mechanism underlying macrophage dysfunction during T2DM pathogenesis.

Exosomes from macrophages are closely linked with various pathogenic conditions such as inflammation and cardiac disorders [37–39]. For instance, exosomes derived from macrophages could repress fibroblast proliferation and accelerate fibroblast inflammation during myocardial infarction-induced cardiac injury [39]. More importantly, macrophage-derived macrophages were also associated with the development of diabetes mellitus. Specifically, exosomes secreted by the macrophages present in adipose tissues regulated the pathogenesis of diabetic obesity through altering the insulin sensitivity of adipocytes [15]. However, the underlying cellular mechanism still remains poorly understood. It has been well-established that the pathogenic progression of T2DM was mediated by the dysregulation of mitochondrial functions in adipocytes such as impairment of mitochondrial CIV complex, which caused abnormal glucose and lipid metabolism [3, 9]. In this study, we showed that exosomes from macrophages under high glucose induced greatly suppressed glucose uptake and impaired mitochondrial CIV complex activity. These observations firmly validated the pathogenic roles of exosomes secreted by macrophages as a response to glucose stimulation during T2DM development.

MicroRNAs (miRNAs) are major components of exosomes and could be transferred to and taken up by neighboring cells or distant cells, thus regulating the functions of recipient cells [40]. In consistence with this, a large number of miRNA molecules have been identified in macrophage-derived exosomes in adipose tissues, which were suggested to be associated with insulin sensitivity and T2DM development [15]. Among these microRNA molecules, miR-210 was highly expressed in adipose tissue macrophages [15]. We showed in this study that miR-210 in exosomes from macrophages was also greatly increased by high glucose, suggesting its possible functions during diabetes development. However, its specific pathogenic and molecular functions during T2DM and obesity are largely unknown. Previous reports also showed that circulating microRNA-210 could be potentially explored as a novel biomarker for T2DM complicated with coronary artery disease [41]. Moreover, the silencing of miR-210 using localized delivery of oligonucleotides was reported to be an effective way of accelerating diabetes-associated chronic wound healing [42]. We clearly showed here that miRNA inhibitors greatly mitigated the effects of macrophage-derived exosomes on adipocyte glucose uptake and mitochondrial respiratory chain activity. These results revealed a new facet of miR-210 functioning during T2DM pathogenesis.

The key roles of miRNAs were mediated by suppressing the expression of target genes via association with their mRNA molecules [43]. Previously, many genes have already been identified as the target genes of miR-210. Specifically, the gene encoding the caspase-8-associated protein 2 was reported to be targeted by miR-210 during the regulation of stem cell survival by ischemic preconditioning treatment [44]. As another example, miR-210, which is highly expressed in the tissues of patients with late stages of lung cancer, was also shown to mediate mitochondrial alterations and changes of HIF-1 (hypoxia-inducible factor-1) activity through targeting the subunit D of succinate dehydrogenase complex (SDH) [45]. However, the target of miR-210 associated with T2DM pathogenesis has not been characterized previously. NDUFA4, formerly known as a constituent of NADH dehydrogenase, was recently identified as a component of the CIV complex associated with mitochondrial dysfunction, syndromic obesity, and the development of diabetes [34–36]. Here, we showed that miR-210 could directly associate with NDUFA4 gene and suppress its expression in adipocytes. The mediating roles of NDUFA4 expression in the regulation of adipocyte glucose uptake and mitochondrial respiratory chain activity were further validated by overexpression of NDUFA4 gene in adipocytes. Together, we convincingly revealed the suppression of NDUFA4 expression by miR-210 in macrophage-derived exosomes mediated the insulin resistance and obesity associated with T2DM pathogenesis.

Finally, the pathogenic roles of miR-210 in diabetes mellitus development have been further verified in this study using a diabetic animal model established using the miR-210 knockout mice. We showed that knockout of miR-210 expression in mice greatly caused significant suppression of diabetic obesity development. Taken together, through the combination of cellular and animal models, we provided in this study that the miR-210 contained in the exosomes derived from the adipose tissue macrophages promote T2DM and obesity development by associating with and suppressing the expression of NDUFA4 gene expression in adipocytes, which provided novel insight into the molecule mechanisms underlying macrophage exosomes and microRNA-mediated diabetes pathogenesis, and might also be explored as a new target for the development of diabetes diagnosis and clinical treatment.

## Figures and Tables

**Table 1:** Sequences of primers used in quantitative RT-PCR assay.

ID		Sequence (5′-3′)	Product length (bp)
*β*-Actin F		CATTGCTGACAGGATGCAGA	139
*β*-Actin R		CTGCTGGAAGGTGGACAGTGA	
NDUFA4	F	TCCCAGCTTGATTCCTCTCTT	117
NDUFA4	R	GGGTTGTTCTTTCTGTCCCAG	
U6	F	CTCGCTTCGGCAGCACA	
U6	R	AACGCTTCACGAATTTGCGT	
All	1R	CTCAACTGGTGTCGTGGA	
miR-210		AGCCACTGCCCACCGCACACTG	
miR-210	RT	CTCAACTGGTGTCGTGGAGTCGGCAATTCAGTTGAGCAGTGTGCG	
miR-210	F	ACACTCCAGCTGGGAGCCACTGCCCACCGCAC	

## Data Availability

The datasets used in this study are available from the correspnding author by reasonable request.
